# Estimation of Cyclic Interstory Drift Capacity of Steel Framed Structures and Future Applications for Seismic Design

**DOI:** 10.1155/2014/496206

**Published:** 2014-06-26

**Authors:** Edén Bojórquez, Alfredo Reyes-Salazar, Sonia E. Ruiz, Amador Terán-Gilmore

**Affiliations:** ^1^Facultad de Ingeniería, Universidad Autónoma de Sinaloa, Calzada de las Américas y Boulevard Universitarios S/N, Ciudad Universitaria, 80040 Culiacán Rosales, SIN, Mexico; ^2^Coordinación de Mecánica Aplicada, Instituto de Ingeniería, Universidad Nacional Autónoma de México, Ciudad Universitaria, 04510 Coyoacán, Mexico; ^3^Departamento de Materiales, Universidad Autónoma Metropolitana, Avenida San Pablo 180, 02200 México, DF, Mexico

## Abstract

Several studies have been devoted to calibrate damage indices for steel and reinforced concrete members with the purpose of overcoming some of the shortcomings of the parameters currently used during seismic design. Nevertheless, there is a challenge to study and calibrate the use of such indices for the practical structural evaluation of complex structures. In this paper, an energy-based damage model for multidegree-of-freedom (MDOF) steel framed structures that accounts explicitly for the effects of cumulative plastic deformation demands is used to estimate the cyclic drift capacity of steel structures. To achieve this, seismic hazard curves are used to discuss the limitations of the maximum interstory drift demand as a performance parameter to achieve adequate damage control. Then the concept of cyclic drift capacity, which incorporates information of the influence of cumulative plastic deformation demands, is introduced as an alternative for future applications of seismic design of structures subjected to long duration ground motions.

## 1. Introduction

Currently, the maximum interstory drift and ductility demands are targeted as response parameters to achieve adequate structural performance of earthquake-resistant structures. Nevertheless, the use of these parameters is not completely justified for buildings subjected to long duration ground motions. In fact, ample evidence suggests that the structural performance of buildings subjected to long duration ground motions is not adequately characterized through maximum deformation demands [1]. Therefore, in some cases, the effect of cumulative plastic deformation demands must be explicitly considered.

Although different energy-based methodologies that aim at providing earthquake-resistant structures with adequate energy dissipating capacity have been proposed [2–5], currently, the most popular response parameter worldwide for seismic design of buildings is the maximum interstory drift. Although the use of energy concepts during practical earthquake-resistant design requires further developments, the influence of cumulative demands should be incorporated into seismic design of structures subjected to long duration motions. A viable manner to account explicitly for cumulative plastic deformation demands is the use of a cyclic (reduced) drift capacity, which in concept is similar to the target ductility concept formulated by [6]. The aim of this paper is firstly to, introduce an energy-based damage index which explicitly accounts for the effects of cumulative plastic deformation demands in steel frames, secondly, to compare demand hazard curves obtained for moment-resisting steel frames in terms of the energy-based damage index and the maximum interstory drift, and finally, to provide for the steel frames drift capacity thresholds (denoted cyclic drift capacity) that account for cumulative damage and that yield adequate levels of reliability.

## 2. Energy-Based Damage Index for Steel Framed Structures

Energy-based methodologies are focused on providing structures with energy dissipating capacities that are equal to or larger than their expected energy demands [2, 7]. The design requirement for an earthquake-resistant structure can be formulated in these terms as

(1) $$\begin{array}{c} \text{Energy}\;\;\text{Capacity}\geq \text{Energy}\;\;\text{Demand}. \end{array}$$

Among all the energies absorbed and dissipated by a structure, the plastic dissipated hysteretic energy, *E*_*H*_, is clearly related to structural damage. *E*_*H*_ can physically be interpreted by considering that it is equal to the total area under all the hysteresis loops that a structure undergoes during a ground motion. Therefore, it is convenient to express (1) in terms of plastic dissipated hysteretic energy:

(2) $$\begin{array}{c} E_{\text{HC}}\geq E_{\text{HD}}, \end{array}$$

where *E*_HC_ is the plastic hysteretic energy capacity and *E*_HD_ is its corresponding energy demand. Equation (2) can be reformulated as an energy-based damage index:

(3) $$\begin{array}{c} I_{\text{DE}}=\frac{E_{\text{HD}}}{E_{\text{HC}}}\leq 1. \end{array}$$

Within the context of (3), the performance level or condition that implies that the energy demand on the system is equal to its corresponding capacity will be considered as the failure of that system. Hence, while *I*_DE_ equal to one corresponds to failure of the structural system, a value of zero implies no structural damage (elastic behavior implies no structural damage). From a physical point of view, this equation represents a balance between the structural capacity and demand in terms of energy. In this sense, this formulation follows the direction initially established by Housner in 1956 for an energy-based design. According to (3), structural damage depends on the balance between the plastic hysteretic energy capacity and demand on the structure. While the plastic hysteretic energy demand can be obtained through dynamic analysis, a challenge exists to define the plastic hysteretic energy capacity of a structure. Nevertheless, flexural plastic behavior is usually concentrated at the ends of the structural members that make up a frame and in the particular case of *W* steel shapes in the flanges. The plastic hysteretic energy capacity of a steel member that forms part of a structural frame can be estimated as follows [2]:

(4) $$\begin{array}{c} E_{\text{HCm}}=2Z_{f}f_{y}\theta_{pa}, \end{array}$$

where *Z*_*f*_ is the section modulus of the flanges, *f*_*y*_ is the yield stress, and *θ*_*pa*_ is its cumulative plastic rotation capacity. Note that the above equation considers that plastic energy is dissipated exclusively through plastic behavior at both ends of a steel member. Equation (4) can be used together with (3) to evaluate the level of structural damage in steel members. However, it is convenient to normalize *E*_*H*_ for damage evaluation purposes [8, 9]:

(5) $$\begin{array}{c} E_{N}=\frac{E_{H}}{F_{y}\delta_{y}}, \end{array}$$

where *F*_*y*_ and *δ*_*y*_ are the strength and displacement at first yield, respectively. Equation (3) can be expressed in terms of *E*_*N*_ as follows:

(6) $$\begin{array}{c} I_{\text{DEN}}=\frac{E_{\text{ND}}}{E_{\text{NC}}}\leq 1, \end{array}$$

where the parameters involved in (6) have similar meanings as those used in (3). The advantage of formulating the problem in terms of *E*_*N*_ is that this is a more stable parameter and thus can be used in quantitative terms for practical purposes. The energy-based damage index proposed herein corresponds to the ratio between the normalized hysteretic energy demand and normalized hysteretic energy capacity, and the condition of failure is assumed to be *I*_DEN_ equal to one. In the case of MDOF steel structures, the principal challenge for the practical use of (6) is the definition of the energy capacity of the structure in terms of that of its structural members. Through the consideration that in regular steel frames the energy is dissipated exclusively by the beams (which is a reasonable assumption for strong column-weak beam structural systems), their energy capacity can be estimated through a modified version of (4) [5]:

(7) $$\begin{array}{c} E_{\text{NC}}=\frac{\sum_{i=1}^{N_{S}}\left(2N_{B}Z_{f}F_{y}\theta_{pa}F_{\text{EH}i}\right)}{C_{y}D_{y}W}, \end{array}$$

where *N*_*S*_ and *N*_*B*_ are the number of stories and bays in the building, respectively, *F*_EH*i*_ is an energy participation factor that accounts for the different contribution of each story to the energy dissipation capacity of a frame, *W* is the total weight of the structure, and, finally, *C*_*y*_ and *D*_*y*_ are the seismic coefficient and displacement at first yield, which are obtained through pushover analysis.

From extensive statistical studies, *F*_EH*i*_ can be estimated as [5]

(8) $$\begin{array}{c} F_{\text{EH}}=\underset{}{\min}\left(F_{\text{EH}}^{*},1\right), \end{array}$$

where

(9) FEH∗=1(−0.0675μ+2.82)h/H×exp⁡{−12[(ln⁡(h/H)−ln⁡(0.031μ+0.3461))0.06μ+0.39]2}.

Equation (7) shows the role of the cumulative plastic rotation capacity of the structural members in the total energy dissipation capacity of a frame. Based on the results of several experimental tests of steel members collected by [10], Bojórquez et al. [11] found that the cumulative plastic rotation capacity of steel members is well represented by a lognormal probability density function with a median value equal to 0.23. Results illustrated below will show that there is no influence of the uncertainty in the selection of the cumulative plastic rotation capacity.

## 3. Maximum Interstory Drift Index

**Figure 1:**
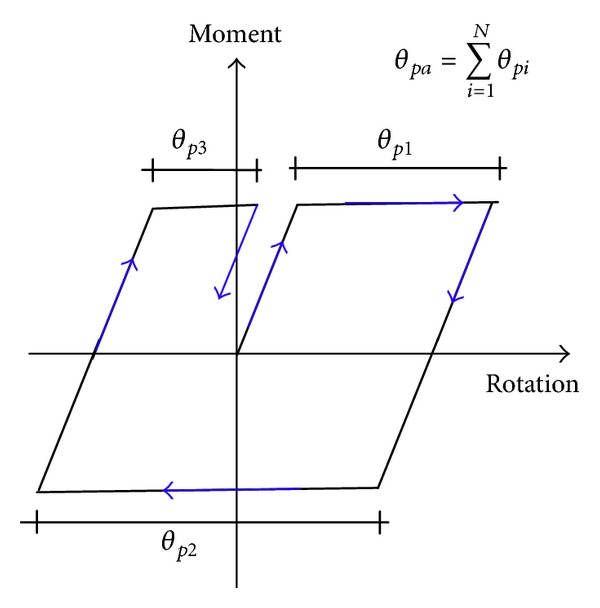
Estimation of cumulative plastic rotation.

The demand hazard curves in terms of maximum interstory drift and the energy-based damage index introduced herein cannot be compared directly unless the maximum interstory drift is normalized by its respective structural capacity. In this manner, a normalized damage measure in terms of interstory drift (denoted interstory drift damage index) needs to be formulated as follows:

(10) $$\begin{array}{c} {I_{D}}_{\gamma }=\frac{\gamma_{D}}{\gamma_{u}}, \end{array}$$

where *I*_*D*__*γ*_ characterizes damage in terms of maximum interstory drift; and *γ*_*D*_ and *γ*_*u*_ represent the demand and capacity of the structure, respectively, in these terms. From pushover analyses of the steel frames under consideration, *γ*_*u*_ was found to be close to 0.05 [11]. Note that while the energy damage index takes into account explicitly the cumulative demands due to plastic deformation, *I*_*D*__*γ*_ is based on peaks demands in such a way that the effect of ground motion duration is not explicitly considered. This can be better explained using the definition of cumulative plastic rotation which is one of the most important parameters to estimate the energy-based damage index and it is schematically illustrated in Figure 1.

## 4. Steel Moment Resisting Frames and Earthquake Ground Motion Records

### 4.1. Steel Moment Resisting Frames

**Figure 2:**
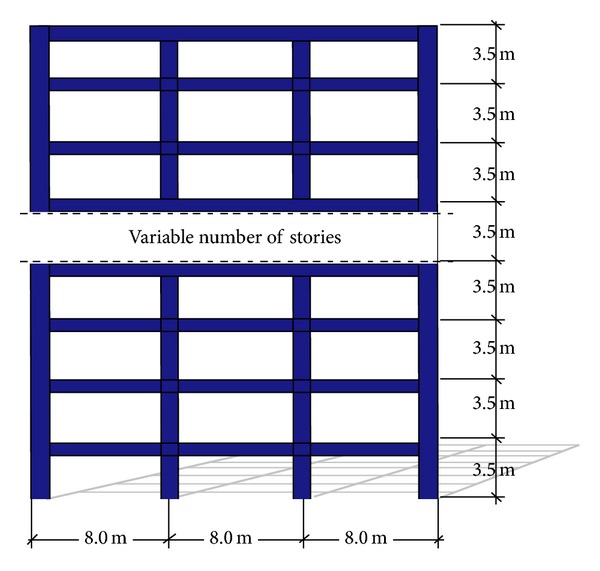
Geometrical characteristic of the MDOF steel frames.

Six moment resisting steel frames having 4, 6, 8, 10, 14, and 18 stories were considered for the studies reported herein. The frames are denoted as F4, F6, F8, F10, F14, and F18, respectively. As shown in Figure 2, the frames (designed according to the Mexico City Building Code, MCBC) have three- to eight-meter bays and interstory heights of 3.5 meters. Each frame was provided with ductile detailing and its lateral strength was established according to the MCBC. A36 steel was used for the beams and columns of the frames. An elastoplastic model with 3% strain-hardening was used to model the cyclic behavior of the steel elements. As discussed by Bojórquez and Rivera [12], this model provides a good approximation to the actual hysteretic behavior of steel members. The columns in the first story were modeled as clamped at their bases. Second order effects were explicitly considered, and 3% of critical damping was used for the two first modes of the frames during the nonlinear dynamic analyses. Relevant characteristics for each frame are summarized in Table 1. In this table, *T*_1_ denotes fundamental period of vibration, *C*_*y*_ denotes seismic coefficient at yield, and *D*_*y*_ denotes roof displacement at yield.

### 4.2. Seismic Records

**Figure 3:**
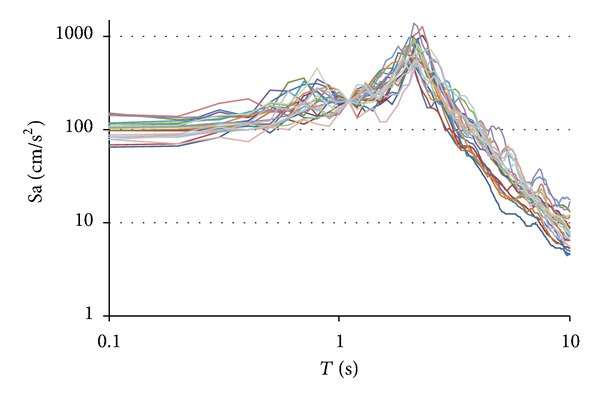
Elastic response spectra for records scaled up to the same value of Sa(*T*_1_) for *T*_1_ of 1.2 sec (3% of critical damping).

A set of 23 narrow-band ground motions recorded at Lake Zone sites of Mexico City was considered. Particularly, all motions were recorded at sites having soil periods of two seconds during seismic events with magnitudes of seven or larger and having epicenters located at distances of 300 km or more from Mexico City. Some important characteristics of the records are summarized in Table 2. While PGA and PGV denote the peak ground acceleration and velocity, respectively, the duration was estimated according to Trifunac and Brady [13]. The seismic records under consideration were established by rotating both horizontal components of a motion recorded at a given station so that its Arias intensity [14] was maximized. The narrow-band records exhibit similar values of parameter *N*_*p*_, which is an indicator of the characteristics of their spectral shape [15]. As a result, there is a strong similarity between their spectral shapes. This is illustrated in the log-log plot included in Figure 3, which shows response spectra of all records scaled to the same spectral acceleration for a period of 1.2 sec (fundamental period of vibration of frame F8). The similitude exhibited by all spectra indicates that the spectral acceleration is a good indicator of the damage potential of the ground motions and emphasizes the good correspondence that exists between parameter *N*_*p*_ and the spectral shape.

## 5. Seismic Vulnerability Assessment

One of the main objectives of earthquake engineering is to quantify, through the consideration of all possible earthquake ground motion intensities at a site, the seismic reliability implicit in structures. Probabilistic seismic demand analysis (PSDA) is used as a tool for estimating the reliability of structures through the evaluation of the mean annual frequency of exceeding a specified value of an earthquake demand parameter EDP (e.g., interstory drift, normalized plastic hysteretic energy, etc.). Based on past studies [16, 17] and considering the total probability theorem, a probabilistic seismic demand analysis can be carried out through the consideration of the mean annual rate of exceeding a given value of EDP:

(11) λEDP(x) =∑νi∫IM∫M∫RP[EDP>x ∣ IM,M,R] ×f(IM ∣ M,R)f(M,R)dr dm d(im),

where *λ*_EDP_(*x*) is the mean annual frequency of EDP exceeding the value *x*, *ν*_*i*_ is the rate of earthquakes for source *i*, *f*(IM∣*M*, *R*) is the conditional distribution function of the intensity measure (IM) given values of magnitude (*M*) and distance (*R*), *f*(*M*, *R*) is the joint probability density function of *M* and *R*, and, finally, *P*[EDP > *x*∣IM, *M*, *R*] is the probability of EDP exceeding *x* given IM, *M*, and *R* (if *x* corresponds to the capacity of the structure, this term represents the fragility curves of the system). If *P*[EDP > *x*∣IM, *M*, *R*] = *P*[EDP > *x*∣IM], then the IM is said to be* sufficient* [18, 19] since its ability to predict the structural response is independent of *M* and *R*, given IM. It has been shown that thespectral acceleration at first mode of vibration Sa(*T*_1_) is* sufficient* with respect to magnitude and distance [19]. As suggested before, the records used herein allow the use of a scaling criteria based on Sa(*T*_1_): (a) first, due to sufficiency of Sa(*T*_1_) with respect to *M* and *R*, (b) second, due to the similar spectral shape of the records, and (c) third, because no bias in nonlinear structural response is observed for different scaling levels of the records under consideration [15]. Within this context, (11) can be expressed as

(12) $$\begin{array}{c} \lambda_{\text{EDP}}\left(x\right)=\overset{}{\underset{\text{Sa}\left(T_{1}\right)}{\int }}P\left[\text{EDP}>x\;|\;\text{Sa}\left(T_{1}\right)=\text{sa}\right]d\lambda_{\text{Sa}(T_{1})}\left(\text{sa}\right), \end{array}$$

where *dλ*_Sa(*T*_1_)_(sa) = *λ*_Sa(*T*_1_)_(sa) − *λ*_Sa(*T*_1_)_(sa + *d*sa) is the differential of the ground motion hazard curve expressed in terms of Sa(*T*_1_). Equation (12) was used to evaluate the structural reliability of the steel frames in terms of two EDPs: interstory drift index and energy damage index. A lognormal distribution is considered to evaluate *P*[EDP > *x*∣Sa].

## 6. Incremental Dynamic Analysis and Fragility Curves

**Figure 4:**
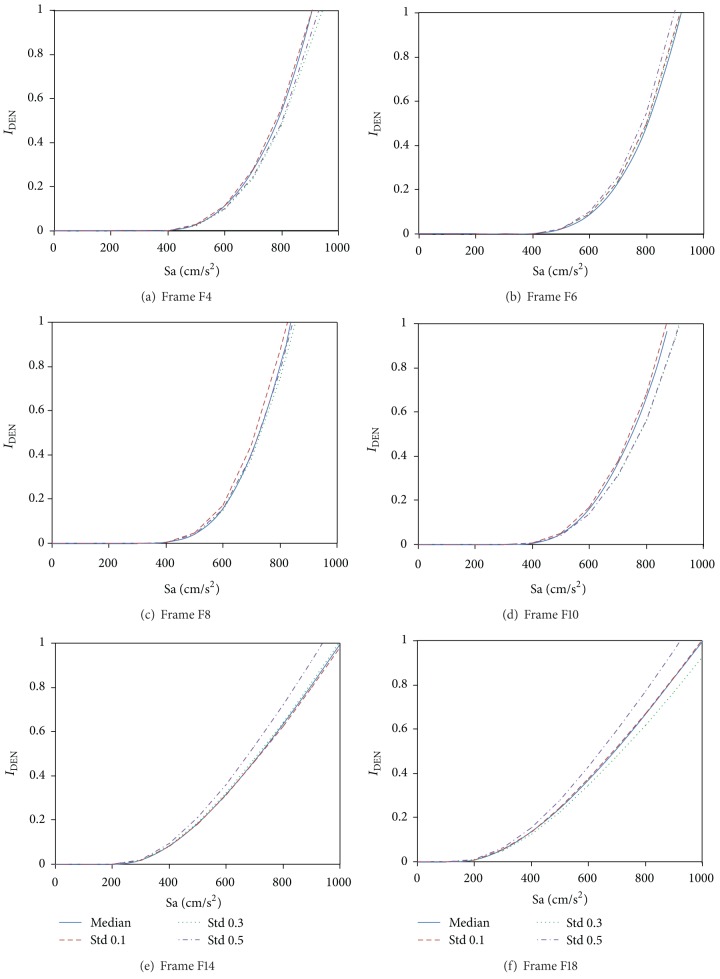
Estimation of *I*_DEN_ at different intensity levels for the frames considering uncertainties in the value of *θ*_*pa*_.

The first step to assess the seismic vulnerability is the estimation of structural demands in terms of both damage measures under consideration at different intensity levels of the spectral acceleration (incremental dynamic analysis). For the sake of brevity only results for the energy-based damage index are discussed and incorporated into the paper since for this damage measure it is important to observe the influence of the uncertainty in the use of *θ*_*pa*_. For this aim, the structural damage in the frames under consideration was computed to assess the effect of explicitly considering the uncertainty of *θ*_*pa*_ of the structural members. A lognormal probability density function with a median value of 0.23 was used to describe the variation of the cumulative plastic rotation capacity at the ends of the beams. For illustrative purposes, four standard deviations of the natural logarithm were considered: 0, 0.1, 0.3, and 0.5. A standard deviation of zero corresponds to the mean values. While it was assumed that the value of *θ*_*pa*_ varied in height according to the lognormal density function, the value of *θ*_*pa*_ for all beams within a story was considered equal. The influence of the uncertainty of *θ*_*pa*_ is illustrated through the results obtained from incremental dynamic analyses of all frames under consideration. For this purpose, the frames were subjected to the ground motions included in Table 2 scaled to achieve the same spectral ordinate at the period corresponding to the first mode of vibration of each particular frame. A wide range of motion intensities were considered for this purpose.  Figure 4 compares the median values of *I*_DEN_ for the steel frames. The horizontal axis considers the different intensity levels quantified through the spectral acceleration associated to the first mode of vibration. The comparison suggests that there is no significant influence of the level of uncertainty of *θ*_*pa*_ in the damage estimates for the frames. In general, the damage estimates corresponding to the different levels of uncertainty are quite similar for each particular frame. It can be concluded that it is reasonable to estimate the structural damage through the consideration of median cumulative rotation capacities and moreover to compute *P*[EDP > *x*∣Sa] for the damage measure under consideration.

**Figure 5:**
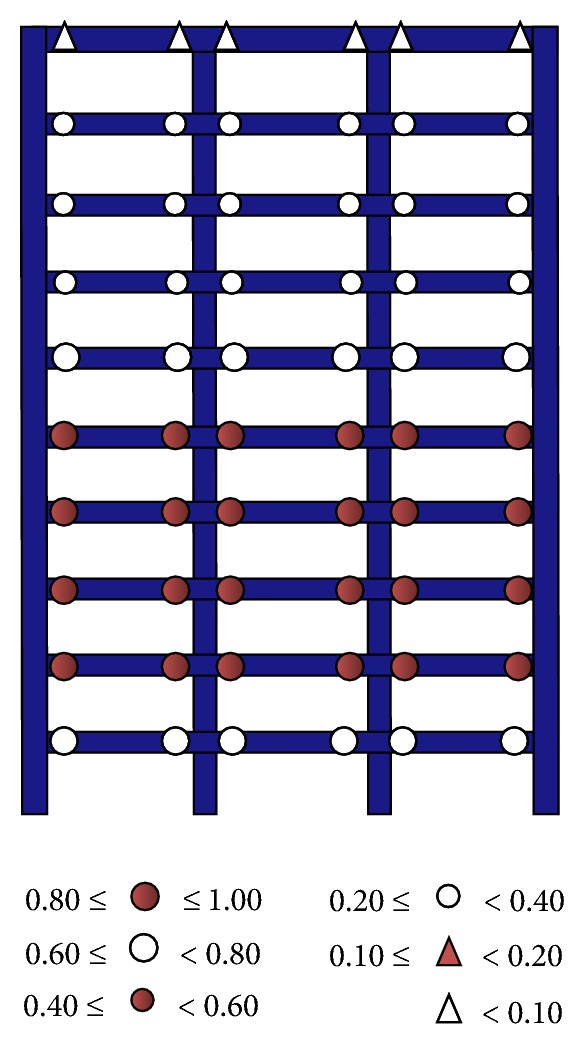
Damage distribution in frame F10, *I*_DEN_ = 1.0.

In Figure 5 the damage distribution of frame F10 in terms of *I*_DEN_ is illustrated. It can be observed that the level of damage in all the beams within a story is practically the same, which is very important to represent the hysteretic energy capacity in a steel frame through (7). As it was discussed before, a value of *I*_DEN_ equal to one theoretically implies structural failure at the critical stories of the frame. Note that according to the figure, the beams located from the second to the fifth story have the largest level of damage which is equal to one. For this reason, it can be concluded that *I*_DEN_ is a useful global parameter that correlates very well with the level of damage in the critical stories of steel frames.

The second step before computing the mean annual rate of exceeding the structural demands is the estimation of fragility curves. By considering a lognormal distribution to assess *P*[EDP > *x*∣Sa], the probability that EDP exceeds *x* given Sa(*T*_1_) is given by

(13) P(EDP>x ∣ Sa(T1)=sa)  =1−Φ(ln⁡x−μ^lnEDP ∣ Sa(T1)=saσ^lnEDP ∣ Sa(T1)=sa).

**Figure 6:**
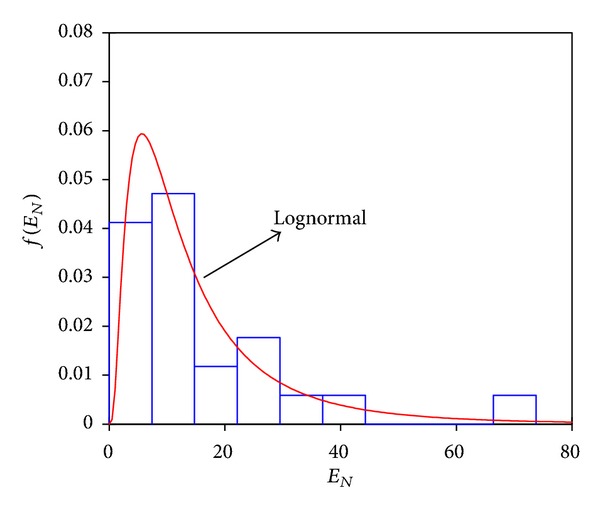
Probability density function for *E*_*N*_ demands of steel frame F10 at a spectral acceleration equal to 1200 cm/s².

In (13), ${\hat{\mu }}_{\text{lnEDP}|\text{Sa}(T_{1})=s_{a}}$ and ${\hat{\sigma }}_{\text{lnEDP}|\text{Sa}(T_{1})=s_{a}}$ are the sample mean and standard deviation for the EDP, respectively, and Φ(·) is the standard normal cumulative distribution function. Although the maximum interstory drift has been found to be well represented by a lognormal distribution [19], a Kolmogorov-Smirnov test was developed to validate the use of this distribution for the case of the normalized plastic hysteretic energy, *E*_*N*_, defined as

(14) $$\begin{array}{c} E_{N}=\frac{E_{H}}{C_{y}D_{y}W}, \end{array}$$

where *E*_*H*_ is the total plastic hysteretic energy dissipated by the structure during the ground motion, *D*_*y*_ and *C*_*y*_ are the global displacement and seismic coefficient at first yield, and *W* is the total weight of the structure. Note that the product of the seismic coefficient times *W* is equal to the base shear of the structure at first yield.  Figure 6 compares the actual *E*_*N*_ probability density function corresponding to frame F10 and Sa(*T*_1_) = 1200 cm/sec², with its analytically derived lognormal density function. The* K*-*S* test for the data set suggests that *E*_*N*_ is well represented by the lognormal probability density function. Similar results have been obtained for other frames and a wide range of values of spectral acceleration. For this reason fragility curves are obtained by considering a lognormal probability density function for both damage measures here selected.

## 7. Cyclic Interstory Drift Capacity for Steel Moment Resisting Frames

**Figure 7:**
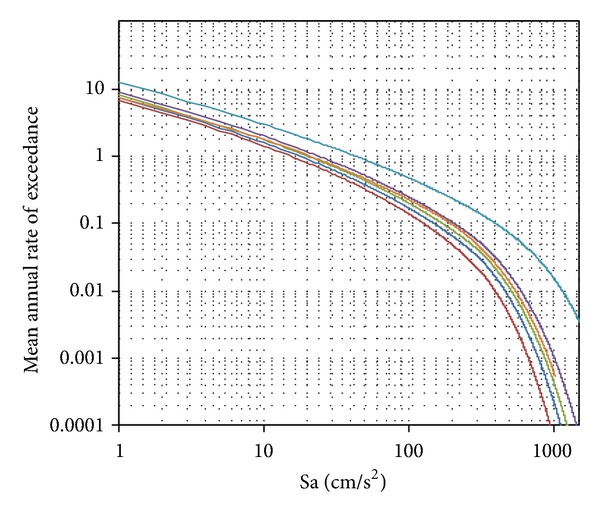
Seismic hazard curves under consideration.

In this study, structural reliabilities were established through the use of (12) and the ground motion seismic hazard curves corresponding to the* Secretaria de Comunicaciones y Transportes* (SCT) site in Mexico City and established by Alamilla [20], which are illustrated in Figure 7 for all the periods of the selected structural steel frames. It can be observed that the curve with the largest mean annual rate of exceeding a spectral acceleration is the blue line which was obtained for a period close to 2 seconds (the structural period of the frame F14). This result was obtained because the soil at the site exhibits a period of two seconds, which is considered representative of the sites where the ground motions under consideration were recorded.

**Figure 8:**
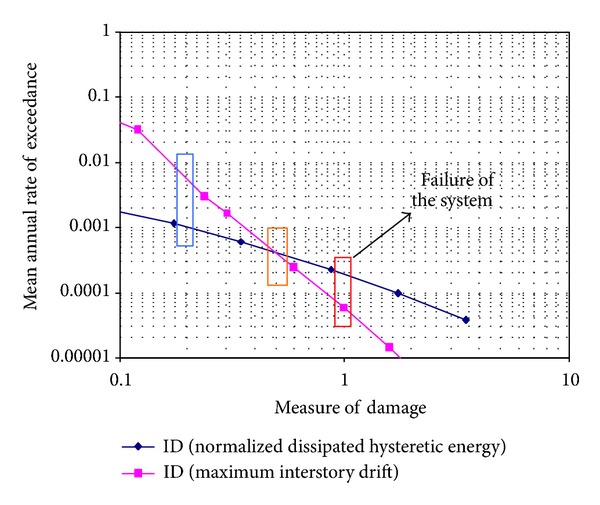
Hazard curves in terms of *I*_DEN_ and *I*_*D*__*γ*_ for frame F4.

A *θ*_*pa*_ = 0.23 was used to characterize the normalized plastic hysteretic energy capacity of the beams as it was previously discussed.  Figure 8 summarizes the demand hazard curves in terms of *I*_*D*__*γ*_ and *I*_DEN_ for frame F4 (values larger than one were plotted for illustrative purposes). Three zones can be appreciated in the figures. The first one corresponds to small values of *I*_*D*_ (blue box), which would commonly be associated to the serviceability limit state. In this range of *I*_*D*_, the mean annual rate of exceedance is larger for *I*_*D*__*γ*_ than for *I*_DEN_. This seems logical since the level of displacement control required by serviceability implies minimum or no plastic demands in the structural elements. A second zone, corresponding to intermediate values of *I*_*D*_ (close to 0.5), can be noticed in Figure 8 (orange box). In this zone, both demand hazard curves exhibit similar ordinates, implying that design for intermediate levels of damage is not sensible to the measure of damage used to guarantee an adequate performance of the frame. Finally, a third zone (red box) can be appreciated for values of *I*_*D*_ close to one. Because this zone relates to failure, it is usually deemed as the most important in terms of practical seismic design. The results suggest that under some circumstances, an unsatisfactory design can be obtained if measures of the ground motion duration or of cumulative demands are not taken into account explicitly. This discussion is valid for all the frames under consideration.

Because in most seismic design codes the principal parameter to promote adequate structural performance is the maximum interstory drift index, it is important to offer maximum interstory drift thresholds that consider the effect of cyclic cumulative deformation (denoted cyclic interstory drift capacity, *γ*_CC_). These thresholds should be established in such manner that the structural reliabilities associated to the failure of the frames are similar in terms of both damage indices used herein.  Table 3 summarizes the reduced interstory drift thresholds that account for energy demands. Note that the maximum reduction in terms of drift threshold occurs for the frames whose fundamental period of vibration is close to two seconds (period of the soil at the site). Technical Requirements for Seismic Design of the MCBC considers a threshold of 0.03 for seismic design of ductile steel frames. This threshold is conservative if compared to the 0.05 value estimated for the frames under consideration. Nevertheless, the cyclic interstory drift ratio thresholds included in Table 3 indicate that the 0.03 threshold may not be conservative enough for structures located in the Lake Zone, particularly for those whose period of vibration is similar to that of the soil. In the latter case, a threshold or cyclic drift capacity of 0.02 would seem more appropriate. To better understand the point of view of the authors notice that the annual rate of exceeding the energy-based damage index equal to 1 is about 0.0002. Hence, the damage index for maximum interstory drift at an annual rate of exceedance of 0.0002 is about 0.65. Since the damage index for maximum interstory drift is defined as the drift demand divided by 0.05, the cyclic interstory drift capacity for F4 is computed using the product of 0.65 and 0.05 (see Table 3).

## 8. Toward Seismic Design of Steel Frames Structures Using an Energy-Based Damage Index by means of the Cyclic Interstory Drift

Because it provides a simple manner to estimate the maximum seismic demands on earthquake-resistant structures, one of the most important tools for seismic design is a well formulated seismic design spectrum. Nevertheless, several issues should be taken into consideration when formulating appropriate design spectra within a code format. A first issue that will be discussed herein is the inclusion of the effect of cumulative deformation demands during the seismic design of an earthquake-resistant structure. In this paper, cumulative demands are explicitly contemplated through the formulation of a damage index that makes explicit consideration of the cumulative plastic deformation demands. It was observed that the use of this energy-based damage index within a design context results in reasonable estimates of structural damage in regular steel frames. Within this context, energy spectra can be used for structural evaluation of complex structures and thus constitute a relevant tool for structural assessment. Note that the energy-based evaluation was formulated in terms of the energy dissipation capacity of earthquake-resistant structures, in such a way that once this capacity is established by using information provided from experimental testing of steel elements, the only other information required in the form of an energy demand can be derived from normalized hysteretic energy spectra.

A second issue that will be addressed is the inclusion of specific reliability levels associated to the structures to be designed. Regarding this, Bojórquez et al. [5] proposed an earthquake-resistant evaluation procedure for steel frames that considers the use of normalized hysteretic energy spectra with uniform annual failure rates. The combination of the energy-based evaluation procedure developed in this paper with the formulation of energy spectra with uniform annual failure rates constitutes itself an excellent alternative to incorporate cumulative demands within specific structural reliability settings.

Finally, this study shows a simplified way for taking into account cumulative damage in steel structures by means of the cyclic interstory drift. The application of this parameter is easier than the use of the energy approach, which is an alternative for a seismic design of steel framed structures that considers cumulative demands. For the case of structures subjected to long duration ground motion and having similar characteristics as those under consideration herein, a reduction of about 30% in the maximum allowable interstory drift is recommended to incorporate the effect of cumulative plastic deformation demands.

## 9. Conclusions

An energy-based damage model for multidegree-of-freedom steel framed structures that accounts explicitly for the effects of cumulative plastic deformation was used to estimate improved thresholds for the maximum interstory drift. The evaluation of the reduced drift limit, denoted cyclic interstory drift capacity, was based on the use of the energy damage index and was targeted to achieve similar levels of structural reliability at failure of the frames in terms of interstory drift and energy.

For the serviceability limit state, seismic design is controlled by maximum interstory drift demands. Nevertheless, in terms of failure, the results presented herein suggest that under some circumstances an unsatisfactory design can be obtained if measures of the ground motion duration or of cumulative demands are not taken into account explicitly. This is particularly important for structures located in very soft soils and having a fundamental period of vibration close to the dominant period of the soil.

Interstory drift ratio thresholds currently used to promote adequate structural performance during severe ground motions usually yield conservative seismic design. Nevertheless, these thresholds need to be carefully assessed for the seismic design of structures located at sites capable of generating long duration motions. Particularly, a cyclic drift capacity of 0.02 seems a better value to achieve adequate structural performance of ductile steel structures located in the Lake Zone of Mexico City, compared to the current value of 0.03 formulated in current Mexican building codes, which indicates a reduction of about 30% of the actual maximum interstory drift capacity.

## Figures and Tables

**Table 1:** Relevant characteristics of the moment resisting steel frames.

Frame	Number of stories	*T*_1_ (s)	*C*_*y*_	*D*_*y*_ (m)
F4	4	0.90	0.45	0.136
F6	6	1.07	0.42	0.174
F8	8	1.20	0.38	0.192
F10	10	1.37	0.36	0.226
F14	14	1.91	0.25	0.30
F18	18	2.53	0.185	0.41

**Table 2:** Seismic records.

Record	Date	Magnitude	Station	PGA (cm/s²)	PGV (cm/s)	Duration (s)
1	25/04/1989	6.9	Alameda	45.0	15.6	45.34
2	25/04/1989	6.9	Garibaldi	68.0	21.5	73
3	25/04/1989	6.9	SCT	44.9	12.8	65.73
4	25/04/1989	6.9	Sector Popular	45.1	15.3	79.13
5	25/04/1989	6.9	Tlatelolco TL08	52.9	17.3	56.55
6	25/04/1989	6.9	Tlatelolco TL55	49.5	17.3	49.91
7	14/09/1995	7.3	Alameda	39.3	12.2	53.6
8	14/09/1995	7.3	Garibaldi	39.1	10.6	86.8
9	14/09/1995	7.3	Liconsa	30.1	9.62	50.94
10	14/09/1995	7.3	Plutarco Elías Calles	33.5	9.37	77.57
11	14/09/1995	7.3	Sector Popular	34.3	12.5	100.76
12	14/09/1995	7.3	Tlatelolco TL08	27.5	7.8	85.76
13	09/10/1995	7.5	Cibeles	14.4	4.6	83.06
14	09/10/1995	7.5	Córdoba	24.9	8.6	94.10
15	09/10/1995	7.5	Liverpool	17.6	6.3	104.95
16	09/10/1995	7.5	Plutarco Elías Calles	19.2	7.9	104.44
17	11/01/1997	6.9	CU Juárez	16.2	5.9	62.09
18	11/01/1997	6.9	Centro urbano Presidente Juárez	16.3	5.5	60.71
19	11/01/1997	6.9	García Campillo	18.7	6.9	84.89
20	11/01/1997	6.9	Plutarco Elías Calles	22.2	8.6	56.34
21	11/01/1997	6.9	Est. # 10 Roma A	21.0	7.76	76.09
22	11/01/1997	6.9	Est. # 11 Roma B	20.4	7.1	74.06
23	11/01/1997	6.9	Tlatelolco TL55	13.4	6.5	55.37

**Table 3:** Cyclic interstory drift capacity.

Frame	*T*_1_ (s)	γ_CC_
F4	0.90	0.032
F6	1.07	0.029
F8	1.20	0.026
F10	1.37	0.023
F14	1.91	0.018
F18	2.53	0.023
